# Efficacy and Safety of Honey Dressings in the Management of Chronic Wounds: An Updated Systematic Review and Meta-Analysis

**DOI:** 10.3390/nu16152455

**Published:** 2024-07-28

**Authors:** Ying Tang, Lihong Chen, Xingwu Ran

**Affiliations:** 1 Department of Endocrinology & Metabolism, West China Hospital, Sichuan University, Chengdu 610041, China; tangying2012@stu.scu.edu.cn (Y.T.); chenlihong@scu.edu.cn (L.C.); 2 Innovation Center for Wound Repair, Diabetic Foot Care Center, West China Hospital, Sichuan University, Chengdu 610041, China

**Keywords:** honey, wounds and injuries, wound healing, chronic wounds, meta-analysis

## Abstract

Chronic wounds impose a substantial economic burden on healthcare systems and result in decreased productivity. Honey possesses diverse properties, rendering it a promising, cost-effective, and efficacious intervention strategy for the management of chronic wounds. However, the findings are controversial. We have presented an updated and comprehensive systematic review and meta-analysis to evaluate the efficacy and safety of honey dressings in the management of chronic wounds. Nine electronic databases were systematically searched to identify relevant studies published prior to 22 March 2024. A total of eight studies, including 906 individuals that met the inclusion criteria, were incorporated. The findings demonstrated a significant acceleration in wound healing time with honey dressings (MD = −17.13, 95% CI −26.37 to −7.89, *p* = 0.0003) and an increase in the percentage of wound healing (MD = 18.31, 95% CI 8.86 to 27.76, *p* = 0.0001). No statistically significant differences were observed in the healing rate (RR = 2.00, 95% CI 0.78 to 5.10, *p* = 0.15), clearance time of bacteria (MD = −11.36, 95% CI: −25.91 to 3.18, *p* = 0.13) and hospital stay duration. Honey may decrease the VAS score but may increase the incidence of painful discomfort during treatment. The topical application of honey is an effective therapeutic approach for managing chronic wounds, but the quality of the evidence was very low due to the quality of risk of bias, inconsistency, and publication bias, highlighting the necessity for larger-scale studies with adequately powered RCTs to ensure the safety and efficacy of honey dressings in chronic wound healing.

## 1. Introduction

Chronic wound refers to the wound where the normal wound healing process is disrupted and difficult to heal. Clinically, it mostly refers to wounds that have not healed or have not shown a healing trend after a certain period of conventional treatment, and the duration ranges from 4 to 12 weeks [1,2]. Chronic wounds have a substantial global impact, affecting millions of individuals and leading to decreased productivity and diminished quality of life. This has profound implications for both global public health and the economy [3,4]. According to the latest data, chronic wounds affect approximately 10.5 million Medicare beneficiaries in the United States alone, contributing to a decline in quality of life among nearly 2.5% of the total population [5]. Therefore, there is an urgent need for more effective and cost-efficient approaches to timely wound treatment and management.

Honey is a viscous, highly concentrated sugar solution derived from the collection, modification, and storage of nectar by honeybees. It primarily comprises approximately 80% carbohydrates (35% glucose, 40% fructose, and 5% sucrose) and 20% water. Additionally, it contains various other constituents such as amino acids, vitamins, minerals, and enzymes [6]. Throughout ancient times, honey has been acknowledged for its medicinal applications. Civilizations such as the Egyptians, Chinese, Greeks, and Romans employed honey in conjunction with herbal remedies to address wound healing. The emergence of resistant strains and the economic burden associated with contemporary dressings have revitalized the utilization of honey as a cost-effective dressing option in developing nations. Many studies have consistently demonstrated the potential of honey in expediting wound healing across various wound types [7,8,9,10,11]. Honey facilitates angiogenesis, granulation tissue formation, and epithelialization to enhance the healing process [12,13]. Previous meta-analyses have explored the therapeutic applications of honey in wound care, highlighting its potential benefits in managing chronic wounds [14]. Despite these insights, existing reviews often exhibit limitations. Most of these studies did not meet the characteristics of chronic wound duration, which may lead to inaccurate results of chronic wounds. Meanwhile, given that China is a major producer of diverse honey varieties and has a long history of using honey in traditional medical practices, it is evident that a significant body of relevant Chinese research might have been overlooked. Therefore, it is crucial to incorporate qualified Chinese literature into systematic evaluations in order to comprehensively evaluate the efficacy of honey in treating chronic wounds. Based on the progress of research, we conducted a comprehensive systematic review and meta-analysis to summarize the updated evidence on the efficacy and safety of honey dressings for the treatment of chronic wounds in RCTs.

The purpose of this meta-analysis was to systematically evaluate the efficacy and safety of honey dressings compared to alternative wound dressings for managing chronic wounds, aiming to provide a comprehensive foundation and reference for clinical decision-making.

## 2. Materials and Methods

The systematic review and meta-analysis of randomized controlled trials (RCTs) were conducted in accordance with the guidelines outlined in the Cochrane Handbook [15] and adhered to the Preferred Reporting Items for Systematic Reviews and Meta-Analyses (PRISMA) statement [16]. The systematic review was prospectively registered in PROSPERO (CRD42023473545).

### 2.1. Search Strategies

A comprehensive computerized search was conducted across multiple databases, including PubMed, Embase, Web of Science, The Cochrane Central Register of Controlled Trials (CENTRAL), ProQuest Dissertations & Theses Global, China National Knowledge Infrastructure (CNKI), Chinese Biomedical Literature service system (SinoMed), Wan Fang Data, and VIP databases from the inception of each database until 22 March 2024. Additionally, we meticulously reviewed the reference lists of all relevant studies and reviews. The detailed search strategy can be found in Table S1.

### 2.2. Eligibility Criteria

This study’s inclusion criteria were established based on the PICOS principles [15]. Participants encompassed a diverse range of patients with chronic wounds, such as diabetic foot ulcers, pressure ulcers, leg ulcers (venous and arterial), surgical wounds healing by secondary intention, and other forms of chronic wounds. The minimum duration of wound existence before intervention was set at ≥4 weeks. The intervention involved the application of honey dressings, while the comparison group received alternative dressings. The primary outcomes assessed in this study were as follows: (a) mean time to achieve wound healing; (b) rate of complete wound healing; and (c) incidence of adverse events. The secondary outcomes included the following: (d) percentage of wound healing (%); (e) assessment of pain intensity during treatment using the Visual Analog Scale (VAS); (f) duration for bacterial clearance in the wound; and (g) length of hospital stay (LOS). The study design employed RCTs. Studies that met any of the following exclusion criteria were not included in the analysis: (i) case reports, animal and in vitro experiments, conference abstracts, letters, case-control studies, self-control studies; (ii) wounds suspected or confirmed to be malignant; (iii) wound duration before treatment was less than 4 weeks or the available data provided in the literature could not determine whether it exceeded 4 weeks; and (iv) studies lacking essential information or original data.

### 2.3. Study Selection and Data Extraction

Two investigators independently screened titles and abstracts of potentially relevant trials, followed by retrieval and assessment of the full text for all relevant or potentially relevant trials pertaining to the review topic. Disagreements were resolved through deliberation to achieve a consensus decision. All data from included articles were extracted and summarized using a standardized format for evaluating study quality and synthesizing evidence. The extracted data encompassed information such as the title, authors’ names, year of publication, and country of origin; characteristics of the studied population, including age, wound type, and sample size; details regarding intervention and comparison groups along with the follow-up period; as well as reported outcomes of interest. For continuous variables, if the standard deviation (SD) value were not provided in the literature, estimate the SD value from *p*-values as per the methods in the Cochrane Handbook. Two researchers independently conducted data extraction while any disagreements were resolved through discussion.

### 2.4. Risk of Bias Assessment

The quality of the included studies was independently assessed by two researchers using the Cochrane’s Risk of Bias (ROB) 2.0 tool [17]. Domains of bias assessment encompass the randomization process, deviations from intended interventions, missing outcome data, measurement of the outcome, and selection of the reported result. Disagreements were resolved through discussion.

### 2.5. Certainty of the Evidence

The Grading of Recommendations, Assessment, Development, and Evaluation (GRADE) approach was used to assess the certainty of evidence by two researchers [18]. The process involves assessing factors such as study design, risk of bias, inconsistency, indirectness, imprecision, and publication bias. Each outcome was evaluated for certainty of evidence and assigned a grade of “high”, “moderate”, “low”, or “very low” based on these criteria. 

### 2.6. Statistical Analysis

The meta-analysis was conducted using RevMan 5.4, with Mean Difference (MD) and 95% confidence intervals (95% CI) reported for continuous outcomes and Risk Ratio (RR) and 95% confidence intervals (95% CI) reported for dichotomous variables. Heterogeneity among trials was assessed using the Chi-square test, while *I*^2^ statistics were used to quantify inconsistency. If *I*^2^ < 50% and *p* > 0.1, data were combined using a fixed effects model. Otherwise, data were performed using a random effects model. Additionally, Stata version 17.0 was utilized to perform sensitivity analyses through leave-one-out meta-analyses in order to evaluate the significance of estimated effects when excluding each study from the meta-analyses. Funnel plots were also conducted to identify potential publication bias. A *p*-value ≤ 0.05 was considered statistically significant.

## 3. Results

### 3.1. Search and Selection of Studies

**Figure 1:**
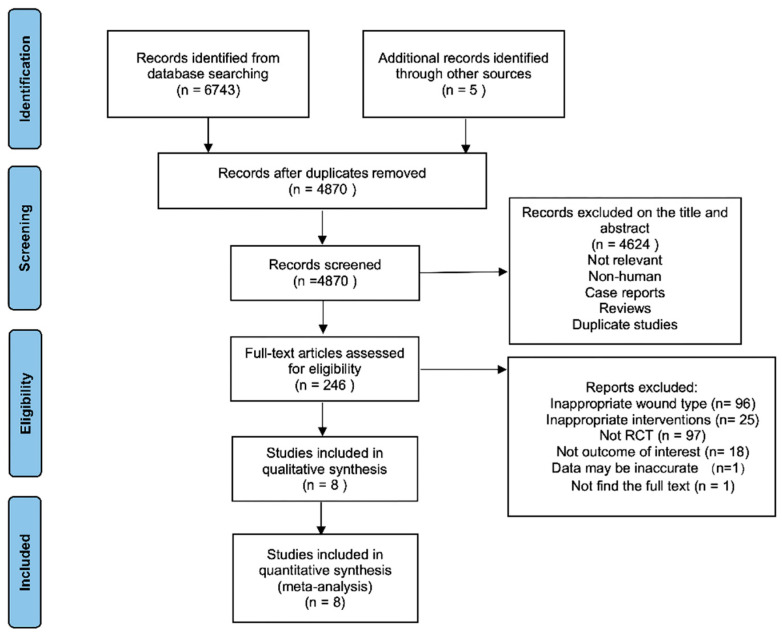
A flow diagram describing the process of selecting studies.

A total of 6682 potential studies were identified, out of which 246 underwent a comprehensive review. Ultimately, a total of eight studies meeting the inclusion criteria were included in the analysis. The process of study identification is visually depicted in Figure 1.

### 3.2. Study Characteristics

The characteristics of these studies are summarized in Table 1. All RCTs were published between 2008 and 2023, with a total enrollment of 906 patients, including 426 in the honey group and 480 in the control group. The included literature encompassed various types of chronic wounds, such as diabetic foot ulcers, venous ulcers, traumatic ulcers, pressure ulcers, and pilonidal cysts, that had a duration exceeding 4 weeks prior to treatment.

### 3.3. Risk of Bias Included Studies

**Figure 2:**
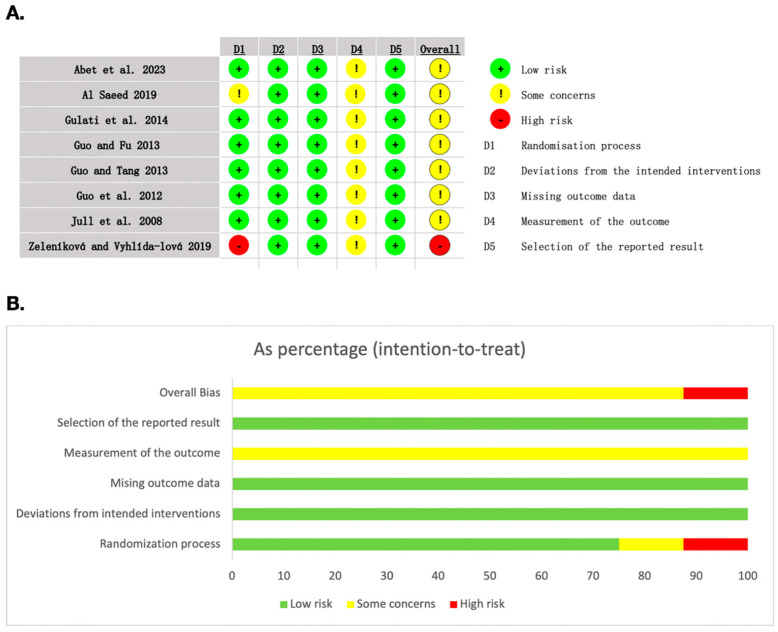
Risk of bias assessment. (**A**) Risk of bias graph. (**B**) Risk of bias summary [7,19,20,21,22,23,24,25].

The methodological quality of the eight included studies is shown in Figure 2. Most studies of randomization have a low risk of bias. Due to honey having a distinctive smell, it was impossible to blind patients and staff during the treatment. However, in the eight studies, such conditions did not lead to deviations from the intended interventions, and the ROB 2.0 algorithm determined these trials had a low risk of bias. However, knowledge of the assigned intervention could influence participant-reported and observer-reported outcomes (such as the level of pain and the assessment of complete healing status). Overall, none of the studies met all ROB 2.0 criteria and were low-risk in all five domains.

### 3.4. Analyses of Outcomes

#### 3.4.1. Mean Time to Achieve Wound Healing

**Figure 3:**
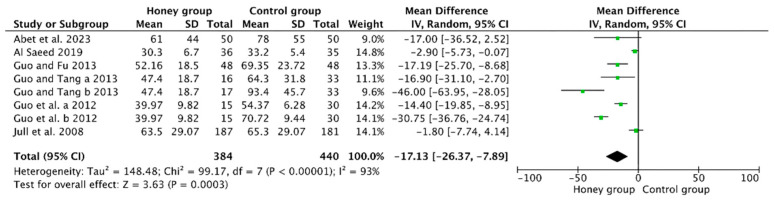
The effect of honey dressing on wound healing time (days) [19,20,21,22,23,24].

The meta-analysis comprised a total of six studies [19,20,21,22,23,24], which collectively demonstrated a significant effect of honey treatment in reducing the average duration for wound healing in chronic wounds (MD = −17.13, 95% CI −26.37 to −7.89, *p* = 0.0003, *I*^2^ = 93%, Figure 3). 

#### 3.4.2. Rate of Complete Wound Healing

**Figure 4:**
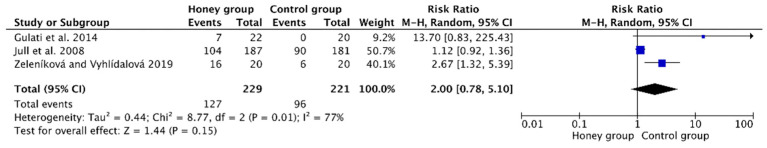
The rate of complete wound healing at the different follow-ups [7,24,25].

The analysis encompassed a total of three studies to evaluate the complete wound healing rate at endpoint [7,24,25]. Meta-analysis findings demonstrated that the honey group exhibited a tendency towards enhancing the rate of complete healing for chronic wounds, although this difference did not reach statistical significance when compared with the control group. (RR = 2.00, 95% CI 0.78 to 5.10, *p* = 0.15, *I*^2^ = 77%, Figure 4).

#### 3.4.3. Incidence of Adverse Events

In the literature examined in this study, Jull (2008) [24] involving 368 participants documented the occurrence of local and systemic adverse effects during honey treatment, whether considered related to the treatment or not. It indicated that treatment with honey was potentially associated with a higher incidence of adverse events (Honey group vs. Control group: 111/187 vs. 84/181, RR = 1.3, 95% CI 1.1 to 1.6, *p* = 0.013), especially in terms of pain (Honey group vs. Control group: 47/187 vs. 18/181, RR = 2.5, 95% CI 1.5 to 4.2, *p* = 0.001). There were no significant differences between the groups for the other adverse events. Gulati (2014) reported that no adverse skin reactions were found during honey treatment [7]. None of the included studies reported possible serious adverse effects from the use of honey dressings.

#### 3.4.4. Percentage of Wound Healing (%)

**Figure 5:**
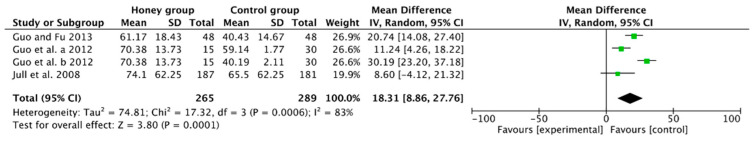
Percentage of wound healing (%) in honey and control group [21,23,24].

The percentage of wound healing was reported in three studies [21,23,24]. Our findings demonstrated a significant improvement in wound healing percentage within the honey group compared to the control group (MD = 18.31, 95% CI 8.86 to 27.76, *p* = 0.0001, *I*^2^ = 83%, Figure 5). 

Gulati (2014) [10] demonstrated that by week six, the surface area of chronic wounds was decreased from 4.25 cm^2^ to 1.95 cm^2^ in the povidone iodine group, while in the honey group, it reduced from 4.35 cm^2^ to 0.55 cm^2^. This difference was significant at 0.05 levels.

#### 3.4.5. The Degree of Pain during Treatment (VAS Scores)

Zeleníková and Vyhlídalová (2019) stated that the VAS scores between two groups were statistically significant at day 20 (Honey group vs. Control group: 1.7 ± 1.53 vs. 3.8 ± 1.7, *p* = 0.0007) [25]. As per Gulati (2014) [7], both groups exhibited a median pain score of 7 at baseline. Following 6 weeks of treatment, the honey dressing group demonstrated a reduction in this score to 1, while the control group experienced a decrease from 7 at baseline to 5 at week six (*p* < 0.001). 

#### 3.4.6. Bacterial Clearance Time of Wounds

**Figure 6:**
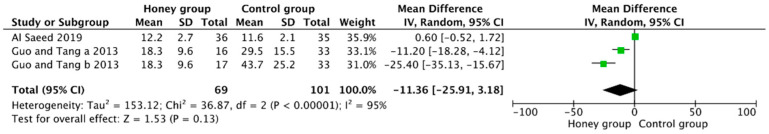
The effectiveness of honey dressings on bacterial clearance time [20,22].

Two studies [20,22] reported the duration required for bacterial clearance in wounds. The findings demonstrated that honey dressings exhibited comparable efficacy to other types of dressings in terms of bacterial removal (MD = −11.36, 95% CI: −25.91 to 3.18, *p* = 0.13, *I*^2^ = 95%, Figure 6).

#### 3.4.7. LOS

Among the included articles, only one study reported the LOS [20]. No statistically significant difference in LOS was observed between the honey group and silver group (13.1 ± 3.5 vs. 12.9 ± 3.2 days, *p* > 0.05).

### 3.5. Sensitivity Analysis

**Figure 7:**
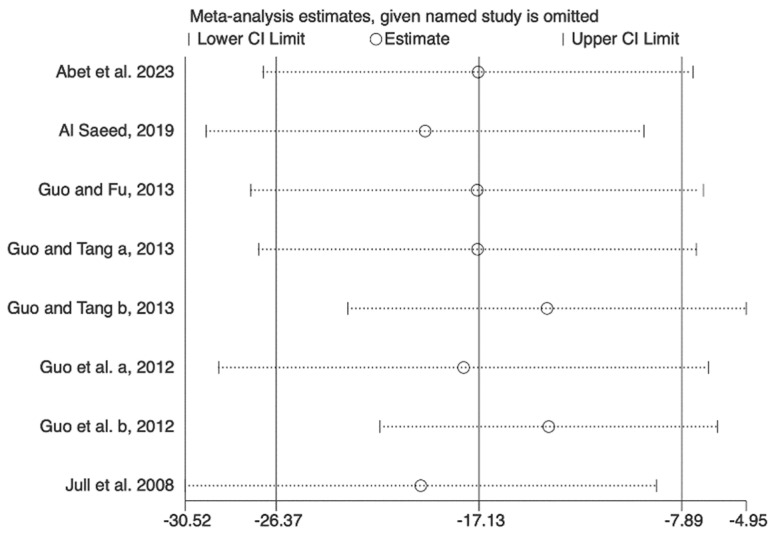
Sensitivity analysis of the effect on mean time to wound healing—leave-one-out analysis [19,20,21,22,23,24].

To evaluate the impact of individual studies on the overall effect sizes, we conducted sensitivity analyses for wound healing time using leave-one-out meta-analyses, systematically excluding one study at a time. However, no significant influence was observed for the outcomes based on leave-one-out analysis (Figure 7).

### 3.6. Publication Bias

**Figure 8:**
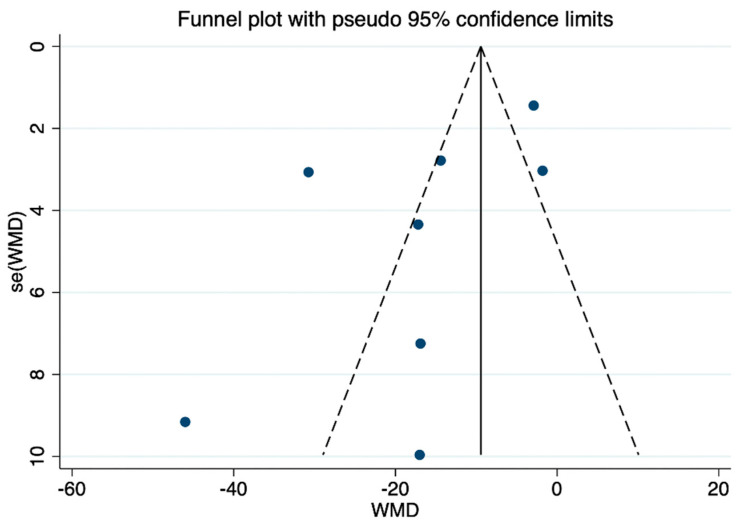
Funnel diagram of mean time to wound healing.

The presence of publication bias in chronic wound healing time was evaluated through the utilization of funnel plots in this meta-analysis. The results indicate a clear asymmetry in the inverted funnel plot, highlighting the necessity to consider potential publication deviations (Figure 8).

### 3.7. Certainty of the Evidence

The findings indicated very low quality as evaluated by the GRADE approach. The significant risk deviations are attributed to the following four factors: (1) some concerns or high risk of attrition bias in included studies; (2) the heterogeneity ≥ 50%; (3) a potential publication bias; and (4) the 95% confidence interval ranges include 1. The detailed results are shown in Table 2.

## 4. Discussion

In this meta-analysis and review, we included a total of eight studies to evaluate the efficacy and safety of honey dressings in the management of chronic wounds. Our findings demonstrate that honey dressings surpass alternative dressings in effectively treating diverse chronic wounds, as evidenced by accelerated wound healing and percentage of wound closure. However, no significant advantages were observed in terms of healing rate, bacterial clearance time and LOS. It is worth noting that the use of honey treatment may decrease the VAS score but may increase the incidence of painful discomfort during treatment. 

Medical-grade honey needs to be gamma sterilized under standardized conditions and free of dangerous microorganisms while preserving its bioactivity [26]. When employed as a dressing, honey establishes an optimal moist environment for wound healing while simultaneously demonstrating rapid antimicrobial activity, deodorizing properties, and mitigating inflammation, edema, and exudation. Research findings have demonstrated the multifaceted effects of honey on wound healing, encompassing antibacterial properties, antioxidant capacity, anti-inflammatory activity, immunomodulatory potential, as well as debridement action and stimulation of wound regeneration [27,28,29]. These pharmacological effects of honey are related to the various bioactive compounds it contains, and these compounds vary in different kinds of honey, thereby influencing their clinical application potential. 

Numerous previous studies have consistently demonstrated the antibacterial properties of honey, which is attributed to its rich composition of natural antibacterial active ingredients, including methylglyoxal (MGO), hydrogen peroxide, polyphenols, antimicrobial peptides, and royal milk main protein. Those compounds have been proposed as significant factors contributing to the antibacterial effects of honey [8,30,31,32]. H_2_O_2_ can cause oxidative damage to biological molecules such as proteins and DNA in bacterial cells, cell membranes, and cytoplasm, thus inhibiting bacterial growth and reproduction [33]. Another type of non-peroxidase antibacterial substance is MGO, which has been found to inhibit both Gram-positive and Gram-negative bacteria [34]. Additionally, antimicrobial peptides like defensin-I and major royal jelly protein-I, found in most types of honey, could directly affect bacteria [28]. Honey’s antibacterial properties are also attributed to its acidity and hyperosmolarity. The pH of honey ranges between 3.2 and 4.5; this low pH inhibits protease activity. Meanwhile, the hyperosmolarity of honey makes the bacteria dehydrated and prevents their proliferation [35]. These two properties of honey create a hostile environment for microbial growth. However, our review revealed that there was no significant difference in the bacterial clearance time of chronic wounds treated with honey dressing compared with other dressings. This lack of statistical significance observed in this study may be attributed to the limited number of included studies, the small sample size within each study, and the high heterogeneity. Further RCTs are warranted to validate the potential efficacy of honey in accelerating bacterial clearance time for wound healing. 

In addition, the antioxidant capacity of honey mainly depends on the components of polyphenols, vitamins, and minerals, which protect cells from oxidative damage by removing reactive oxygen species [36]. In addition, honey can stimulate the production of modulator cytokines TNF-a, IL-1, and IL-6, play a significant anti-inflammatory role, help reduce the inflammatory response around the wound, and have the ability to modulate the activity of immunocompetent cells to promote wound healing [37]. Many clinical trials have shown honey has the ability of debridement, and this effect may be related to the increased activity of fibrinolytic protease [38]. Overall, the various bioactive ingredients of honey work together to show a significant combined effect in preventing infection, reducing inflammation, and thus promoting wound healing. Our study findings provide compelling evidence for the significant acceleration of chronic wound healing through honey dressings, as evidenced by improved wound healing time and percentage.

Our analysis found the honey group may have caused more discomfort during treatment. It has been suggested that the acidic nature of honey may contribute to the perception of pain [28]. We conducted an analysis to explore potential factors that might contribute to these disparities and identified a lack of standardized pain evaluation criteria across the included studies, as well as the subjective nature of pain perception by the patient’s supervisor, which may introduce bias in the assessment process. Additionally, it is crucial to consider the limited reporting of adverse reactions and potential publication bias. Furthermore, pain management should therefore be an integral component of wound care protocols when employing honey dressings, ensuring both patient comfort and optimal healing outcomes are achieved.

Our study has several strengths. Firstly, we have included studies conducted in China that were previously excluded from systematic reviews. Secondly, to ensure accuracy, we have precisely defined chronic wounds as those with a minimum duration of 4 weeks prior to trial entry, excluding self-described ‘chronic wounds’ mentioned in the literature without a specified duration or with durations less than 4 weeks before intervention. Moreover, we incorporated studies with trial durations extended by a minimum of 4 weeks, thereby enhancing the robustness and applicability of our findings for future research endeavors and clinical guidance. In summary, our study presented more compelling evidence.

Our findings should be interpreted within the context of the following study limitations. Firstly, criteria such as the selection of intervention means, frequency, and time of the included studies have not been unified, clinical heterogeneity of the wound etiologies was high, and the sample size of the included studies was small, which made it difficult to conduct subgroup analysis to further find the causes of heterogeneity. Secondly, blinding was not available in the studies, so it may be reducing the reliability of the results of the meta-analysis. In addition, the very low certainty of the evidence seriously affected the estimation of the effectiveness of honey to improve chronic wounds and the confidence in its clinical application. Therefore, future research should prioritize conducting more RCTs with robust study designs to establish the efficacy of honey dressings for treating chronic wounds.

## 5. Conclusions

In conclusion, the application of honey dressings may serve as an efficacy and safety method in managing chronic wounds. However, due to the predominance of very low-quality evidence, the results of this study should be treated with caution, and high-quality literature is imperative to substantiate the efficacy and safety profile of honey dressings for wound healing in the future.

## Figures and Tables

**Table 1:** Characteristics of studies.

Author (Year)	Country	Type of Honey	Sample Size (E/C)	Wound Etiology	Interventions, Frequency and Treatment Duration	Control	Follow-Up Period or Time	Outcome Measure
Abet et al., 2023 [19]	France	NA	50/50	Pilonidal cyst	Honey + alginate dressing; not mention the specific usage	Alginate dressing	180 days	(a)
Al Saeed, 2019 [20]	Saudi Arabia	Manuka honey	36/35	Diabetic foot ulcer	Manuka honey dressing covered with an occlusive secondary one; changed daily or more frequently if the dressing was markedly soaked, until the infection was eradicated and healthy granulations were formed	Controlled release silver hydrophilic dressing	Until healed	(a), (f), (g)
Gulati et al., 2014 [7]	India	Azadericta indica honey	22/20	Various etiology ^a^	Honey was applied to fill the wound cavity sufficiently (1–2 mL) and then covered with film dressing (Tegaderm). Patients with venous leg ulcers were reinforced by elastic compression garments, changed on alternate days for 6 weeks.	Povidone iodine dressing	6 weeks	(b), (c), (d), (e)
Guo and Fu, 2013 [21]	China	Wild native honey in Shennongjia	48/48	Various etiology ^b^	Honey dressing covered the wound (apply directly to the wound up to 0.5 cm thick appropriately). Change the dressing when the outer layer dressing is permeated by seepage > 1/2, until healed or ready for surgical closure	Functional dressing	Until healed or ready for surgical closure	(a), (d)
Guo and Tang, 2013 [22]	China	Dandelion honey	33/66	Diabetic chronic ulcers	Honey dressing covered 3–4 layers (apply directly to the wound up to 0.5 cm thick appropriately). Change the dressing when the outer layer dressing is permeated by seepage > 1/2, until healed or ready for surgical closure	C1: Functional dressing C2: Povidone iodine dressing	Until healed or ready for surgical closure	(a), (f)
Guo et al., 2012 [23]	China	Wild native honey in Shennongjia	30/60	Traumatic skin chronic ulcers	Honey dressing covers 3–4 layers (apply directly to the wound up to 0.5 cm thick appropriately); once daily application initially and then, frequency determined by clinical need, until healed or ready for surgical closure	C1: Functional dressing C2: Conventional dressing	Until healed or ready for surgical closure	(a), (d)
Jull et al., 2008 [24]	New Zealand	Manuka honey	187/181	Venous ulcers	Manuka honey impregnated into calcium alginate dressing + compression bandaging; frequency determined by clinical need	Usual care (received dressings that the district nurse deemed appropriate at the time of each visit)	12 weeks	(a), (b), (c), (d)
Zeleníko vá and Vyhlídalo vá, 2019 [25]	Czech Republic	Manuka honey	20/20	Various etiology ^c^	Honey dressing; not mention the specific usage	Povidone iodine, nanocrystalline silver, or hydrogel	90 days	(b), (e)

Abbreviation: NA: not available. ^a^ Chronic wound of duration ≥6 weeks; ^b^ Diabetic foot ulcer and pressure ulcer; ^c^ non-healing wounds, including pressure ulcers, lower leg ulcers, and diabetic ulcers. (a) Mean time to achieve wound healing; (b) rate of complete wound healing; and (c) incidence of adverse events. The secondary outcomes included the following: (d) percentage of wound healing (%); (e) assessment of pain intensity during treatment using Visual Analog Scale (VAS); (f) duration for bacterial clearance in the wound; and (g) length of hospital stay (LOS).

**Table 2:** GRADE summary of evidence.

Quality Assessment							No. of Patients		Effect		Quality	Importance
No of Studies	Design	Risk of Bias	Inconsistency	Indirectness	Imprecision	Other Considerations	Honey Dressing	Control	Relative (95% CI)	Absolute		
Mean time to achieve wound healing (Better indicated by lower values)												
6	randomized trials	serious ^1^	serious ^2^	no serious indirectness	no serious imprecision	reporting bias ^3^	384	440	-	MD 17.13 lower (from 26.37 to 7.89 and less)	⊕OOO VERY LOW	CRITICAL
Complete wound healing rate												
3	randomized trials	serious ^1^	serious ^2^	no serious indirectness	serious ^4^	reporting bias ^3^	127/229 (55.5%)	96/221 (43.4%)	RR 2 (0.78 to 5.1)	434 more per 1000 (from 96 and less to 1000 and more)	⊕OOO VERY LOW	CRITICAL
								30%		300 more per 1000 (from 66 and less to 1000 and more)		
Percentage of wound healing (%) (Better indicated by lower values)												
3	randomized trials	serious ^1^	serious ^2^	no serious indirectness	no serious imprecision	reporting bias ^3^	265	289	-	MD 18.31 higher (from 8.86 to 27.76 and higher)	⊕OOO VERY LOW	CRITICAL
Bacterial clearance time of wounds (Better indicated by lower values)												
2	randomized trials	serious ^1^	serious ^2^	no serious indirectness	no serious imprecision	reporting bias ^3^	69	101	-	MD 11.36 lower (from 25.91 and lower to 3.18 and higher)	⊕OOO VERY LOW	IMPORTANT

^1^ Downgrading one level for some concerns or high risk of attrition bias in included studies. ^2^ Downgrading one level for the heterogeneity ≥ 50%. ^3^ Downgrading one level for potential publication bias. ^4^ Downgrading one level for the 95% confidence interval ranges from 0.78 to 5.10.

## Data Availability

The original contributions presented in this study are included in the article/Supplementary Material; further inquiries can be directed to the corresponding author.

## Supplementary Materials

The following supporting information can be downloaded at: https://www.mdpi.com/article/10.3390/nu16152455/s1. Table S1: Search Strategy.
