# Supplementary material for: Efficacy and Safety of Honey Dressings in the Management of Chronic Wounds: An Updated Systematic Review and Meta-Analysis
Source: Nutrients. 2024 Jul 28;16(15):2455. doi: 10.3390/nu16152455 (PMC11314015; doi:10.3390/nu16152455)
Supplement: Supplementary file 1 [file nutrients-16-02455-s001.zip › nutrients-3063175-supplementary.pdf]

Table S1. Search Strategy.

Database	Search strategy
Pubmed	(“Wounds and Injuries”[Mesh] OR “wound” [Title/Abstract] OR “ulcer” [Title/Abstract] OR “diabetic foot” [Title/Abstract] OR “diabetic foot ulcer” [Title/Abstract] OR “arterial ulcer” [Title/Abstract] OR “venous ulcer” [Title/Abstract] OR “stasis ulcer” [Title/Abstract] OR “varicose ulcer” [Title/Abstract] OR “pressure ulcer” [Title/Abstract] OR “bedsore” [Title/Abstract] OR “decubitus” [Title/Abstract]) AND (“honey” [Mesh] OR “medical grade honey” [Title/Abstract] OR “honey dressing” [Title/Abstract])
Embase	（'wound'/exp OR ‘wound’:ti,ab,kw OR ‘ulcer’:ti,ab,kw OR ‘diabetic foot’:ti,ab,kw OR ‘diabetic foot ulcer’:ti,ab,kw OR ‘arterial ulcer’:ti,ab,kw OR ‘venous ulcer’:ti,ab,kw OR ‘stasis ulcer’:ti,ab,kw OR ‘varicose ulcer’:ti,ab,kw OR ‘pressure ulcer’:ti,ab,kw OR ‘bedsore’:ti,ab,kw OR ‘decubitus’:ti,ab,kw) AND (‘honey'/exp OR ‘medical grade honey’:ti,ab,kw OR ‘honey dressing’:ti,ab,kw)
CENTRAL	(MeSH descriptor: [Wounds and Injuries] explode all trees OR (wound): ti,ab,kw OR (ulcer): ti,ab,kw OR (diabetic foot): ti,ab,kw OR (diabetic foot ulcer): ti,ab,kw OR (arterial ulcer): ti,ab,kw OR (venous ulcer): ti,ab,kw OR (stasis ulcer): ti,ab,kw OR (varicose ulcer): ti,ab,kw OR (pressure ulcer): ti,ab,kw OR (bedsore): ti,ab,kw OR (decubitus): ti,ab,kw) AND (MeSH descriptor: [Honey] explode all trees OR (medical grade honey): ti,ab,kw OR (honey dressing): ti,ab,kw)
Web of science	(TS=(wound) OR TS=(ulcer) OR TS=(diabetic foot) OR TS=(diabetic foot ulcer) OR TS=(arterial ulcer) OR TS=(venous ulcer) OR TS=(stasis ulcer) OR TS=(varicose ulcer) OR TS=(pressure

	ulcer) OR TS=(bedsore) OR TS=(decubitus)) AND (TS=(honey) OR TS=(medical grade honey) OR TS=(honey dressing) OR TS=(honey-impregnated dressing))
ProQuest Dissertations & Theses Global	TI("wound" OR "ulcer" OR "diabetic foot" OR "diabetic foot ulcer" OR "arterial ulcer" OR "venous ulcer" OR "stasis ulcer" OR "varicose ulcer" OR "pressure ulcer" OR "bedsore" OR "decubitus") OR AB("wound" OR "ulcer" OR "diabetic foot" OR "diabetic foot ulcer" OR "arterial ulcer" OR "venous ulcer" OR "stasis ulcer" OR "varicose ulcer" OR "pressure ulcer" OR "bedsore" OR "decubitus") AND TI("honey" OR "medical grade honey" OR "honey dressing") OR AB("honey" OR "medical grade honey" OR "honey dressing")
